# The significance of calcium ions in cerebral ischemia-reperfusion injury: mechanisms and intervention strategies

**DOI:** 10.3389/fmolb.2025.1585758

**Published:** 2025-05-12

**Authors:** Yong-Wang Li, Yu Liu, Sheng-Zhen Luo, Xiao-Juan Huang, Yan Shen, Wei-Si Wang, Zhi-Chen Lang

**Affiliations:** Department of Anesthesiology, The Third People’s Hospital of Longgang, Clinical Institute of Shantou University Medical College (The Third People’s Hospital of Longgang District Shenzhen), Shenzhen, Guangdong, China

**Keywords:** calcium ions, cerebral ischemia, reperfusion injury, cell death, intervention strategies

## Abstract

Cerebral ischemia-reperfusion injury (CIRI) represents a multifaceted pathological phenomenon characterized by an array of molecular and cellular mechanisms, which significantly contribute to neurological dysfunction. Evidence suggests that calcium ions play an indispensable role in this context, as abnormal elevations in calcium concentrations exacerbate neuronal injury and intensify functional deficits. These ions are integral not only for intracellular signaling pathways but also for various pathological processes, such as programmed cell death, inflammatory responses, and oxidative stress. This review article elucidates the physiological framework of calcium homeostasis and the precise mechanisms through which calcium ions influence CIRI. Moreover, it addresses potential intervention strategies, including calcium channel blockers, calmodulin (CaM) inhibitors, antioxidants, and anti-inflammatory agents. Despite the proposal of certain intervention strategies, their effectiveness and safety in clinical settings warrant further scrutiny. In conclusion, the article highlights the limitations of current research and anticipates future investigative trajectories, aiming to provide a theoretical foundation and reference for the development of more efficacious treatment modalities.

## 1 Introduction

Cerebrovascular disorders pose a significant challenge to human health, characterized by high incidence rates, disability, and mortality, thereby imposing a considerable burden on society and families (Zhang et al., 2024a). A major challenge in the management of ischemic cerebrovascular disorders is Cerebral Ischemia-Reperfusion Injury (CIRI) (Zhang et al., 2024b; Zhu et al., 2025). This condition arises when brain tissue experiences ischemia due to vascular blockages or other factors, making the timely restoration of blood flow through reperfusion essential for recovering brain function. However, in clinical practice, it is not uncommon for brain tissue damage to worsen following reperfusion, a phenomenon referred to as CIRI (Deng et al., 2023; Huang et al., 2024; Ye et al., 2025). The underlying mechanisms of this injury are highly complex, involving a range of physiological and pathological changes, including oxidative stress, inflammatory responses, apoptosis, and autophagy. These processes are interrelated and collectively contribute to the initiation and advancement of the injury (Bai et al., 2018; Li et al., 2024; Liu and Sang, 2024).

Calcium ions are central to understanding the complex mechanisms involved in CIRI (DI et al., 2024; Sharma et al., 2024a; Yaghoobi et al., 2024). As vital intracellular second messengers, they are critical in regulating the normal physiological functions of neurons (Bollimuntha et al., 2011; Ono et al., 2023; Jha et al., 2024). Including maintaining cell membrane potential and facilitating various biochemical reactions within cells. However, during CIRI, the balance of intracellular calcium ions is disrupted, leading to an excessive influx of calcium into the cells (Yue et al., 2017; Dai et al., 2024a; Yang et al., 2025). This calcium overload triggers a cascade of reactions that can damage and ultimately kill nerve cells (Payal et al., 2023; Sharma et al., 2024b; Singh et al., 2025). The consequences extend beyond direct cellular dysfunction; the activation of various enzymes, such as phospholipases, proteases, and nucleases, results in the destruction of cell membranes and organelles, further worsening brain tissue damage. Additionally, calcium overload is intricately linked to other injury mechanisms, including oxidative stress and inflammatory responses, creating a vicious cycle that exacerbates CIRI (Wang et al., 2017; Li et al., 2021a).

In-depth research on the role of calcium ions in CIRI is crucial for understanding the underlying pathophysiological processes and identifying effective therapeutic targets and intervention strategies. By exploring the mechanisms related to calcium ions, researchers aim to establish a foundation for developing new treatment approaches that could enhance patient outcomes, reduce disability and mortality rates, and ultimately improve the quality of life for those affected by cerebral ischemia (Gu et al., 2024). Recently, there has been a growing interest in investigating the role of calcium ions in this context, particularly with the emergence of novel intervention strategies (Kadas et al., 2017; Li et al., 2021b). These include selective calcium channel antagonists, ultrasound therapy, and the use of traditional Chinese medicine, all of which have demonstrated promising neuroprotective effects. Such studies are paving the way for innovative clinical treatments, especially in the prevention and management of CIRI, where maintaining calcium ion homeostasis has become a significant focus of research (Cheung, 2003; Liu and Sang, 2024; Sharma et al., 2024a).

In summary, the role of calcium ions in CIRI is intricate and multifaceted. Gaining a clearer insight into their specific functions within the pathological processes and exploring intervention strategies holds significant clinical importance for improving treatment outcomes and enhancing patient prognosis. Future research should prioritize investigating the involvement of calcium ions in CIRI, as well as identifying potential therapeutic targets. This focus will provide a solid theoretical foundation for the development of innovative therapeutic strategies (Liu et al., 2019; Xie et al., 2021; Yaghoobi et al., 2024).

### 1.1 Overview of cerebral ischemia-reperfusion injury

#### 1.1.1 Definition and current status

CIRI refers to the phenomenon where, after a period of cerebral ischemia, the restoration of blood flow leads to further exacerbation of ischemic damage. It involves a variety of complex pathophysiological processes, including energy metabolism disorders, free radical damage, inflammatory responses, apoptosis, and necrosis. This type of injury is quite common in ischemic cerebrovascular diseases, such as cerebral thrombosis and cerebral embolism. When a cerebral vessel is obstructed, it results in local brain tissue ischemia and hypoxia, during which the metabolism and function of nerve cells are suppressed. If blood flow is restored within a certain timeframe, it is often assumed that the damaged brain tissue can be salvaged; however, the reality is that brain tissue damage often worsens, leading to more severe consequences such as aggravated neurological dysfunction, brain edema, and cell death (Dai et al., 2024b; Ye et al., 2025).

The high harm of this injury is self-evident, as it not only causes a rapid deterioration in the patient’s condition, increasing disability and mortality rates, but also places a heavy burden on the patient’s family and society. Statistics show that a significant proportion of patients with ischemic stroke experience CIRI, severely affecting their prognosis and quality of life (Luo et al., 2023). Many patients may still face long-term neurological deficits, such as limb paralysis, speech disorders, and cognitive decline, even after receiving reperfusion therapy, potentially leading to prolonged bed rest and loss of self-care ability (Wang et al., 2024). Therefore, in-depth research into the pathogenesis of CIRI and the search for effective treatment methods is of utmost urgency and practical significance.

#### 1.1.2 Pathogenesis

##### 1.1.2.1 Free radical damage

Under standard physiological conditions, the body possesses a robust antioxidant defense mechanism that efficiently neutralizes the limited quantities of free radicals produced during metabolic processes, thus maintaining redox balance. However, during cerebral ischemia, tissues experience ischemic and hypoxic conditions, which disrupt mitochondrial respiratory chain functionality and impede electron transfer processes. Such disruptions result in considerable electron leakage, which reacts with oxygen to form superoxide anion free radicals. Simultaneously, ischemia inhibits the synthesis and activity of critical antioxidant enzymes, such as superoxide dismutase (SOD), catalase (CAT), and glutathione peroxidase (GSH-Px), thereby reducing the body’s ability to eliminate free radicals. Consequently, free radicals progressively accumulate, intensifying cellular injury (Chen et al., 2013).

Upon reperfusion, a substantial influx of oxygen enters the previously ischemic tissue, providing an abundant substrate for free radical production. At this juncture, xanthine oxidase catalyzes the oxidation of hypoxanthine and xanthine, leading to the production of uric acid alongside a significant generation of superoxide anion free radicals. Additionally, activated neutrophils and macrophages produce a considerable amount of free radicals, including hydroxyl radicals and hydrogen peroxide, through respiratory bursts. These free radicals exhibit potent oxidative properties, allowing them to target polyunsaturated fatty acids within cell membranes, thereby initiating lipid peroxidation reactions. The byproducts of lipid peroxidation, such as malondialdehyde (MDA), further compromise the structure and functionality of cell membranes, resulting in increased permeability, the leakage of intracellular substances, and the penetration of harmful external agents into the cells. This cascade ultimately leads to cellular damage and death. Moreover, free radicals can oxidize proteins and nucleic acids, altering the structure and functionality of proteins, which affects enzymatic activities and cellular signaling. They can also induce DNA strand breaks and base modifications, resulting in gene mutations and subsequent cellular apoptosis (Sun et al., 2018; Wu et al., 2020).

##### 1.1.2.2 Inflammatory response

During CIRI, the inflammatory response plays a pivotal role in the overall process, which is characterized by complex interactions among various cells and inflammatory mediators. In the initial stages of ischemia, neural and glial cells in the brain are activated by the lack of blood flow and oxygen, leading to the release of inflammatory mediators such as tumor necrosis factor-alpha (TNF-α), interleukin-1 beta (IL-1β), and interleukin-6 (IL-6). These mediators trigger endothelial cells to express adhesion molecules like intercellular adhesion molecule-1 (ICAM-1), P-selectin, and E-selectin. Inflammatory cells, including neutrophils and monocytes, possess receptors for these adhesion molecules, allowing them to adhere to the endothelial surface. Following this adhesion, chemokines facilitate the movement of these inflammatory cells through the endothelial layer into the brain tissue. Once inside, these cells become further activated, releasing additional inflammatory mediators and proteolytic enzymes that can directly harm neural and endothelial cells. This damage disrupts the blood-brain barrier, increases vascular permeability, and can lead to brain edema and hemorrhage. Moreover, the released mediators attract even more inflammatory cells to the injury site, creating a vicious cycle that intensifies the inflammatory damage to the brain. This inflammatory response can also cause disturbances in microcirculation, which diminishes blood flow to the brain, exacerbating the ischemic and hypoxic conditions and promoting the apoptosis and necrosis of neural cells (Yang et al., 2020; Zhuang et al., 2021; Cao et al., 2024).

##### 1.1.2.3 Apoptosis and necrosis

Apoptosis and necrosis are the two primary forms of neuronal cell death observed in CIRI, each with distinct mechanisms and morphological changes, yet both significantly affect brain function. Apoptosis, often described as programmed cell death, is an active process regulated by a series of genes. In the context of CIRI, several factors can trigger apoptosis, including energy metabolism disturbances due to ischemia and hypoxia, free radicals produced from oxidative stress, and inflammatory mediators released during the inflammatory response, all of which can activate intracellular apoptotic signaling pathways (Zhuang et al., 2021). Mitochondria are crucial in this process; a decline in mitochondrial membrane potential leads to the release of cytochrome C into the cytoplasm, where it interacts with apoptotic protease activating factor-1 (Apaf-1) and caspase-9 to form apoptosomes. This complex then activates downstream effector caspases, such as caspase-3, initiating a cascade of apoptotic reactions. Morphologically, apoptosis is characterized by cell shrinkage, chromatin condensation, nuclear fragmentation, and the formation of apoptotic bodies, which are ultimately engulfed and cleared by macrophages. While apoptosis is more prevalent in the early stages of CIRI, it is important to note that despite being a programmed form of cell death, the loss of a significant number of neural cells can lead to severe brain function impairments, including cognitive and motor dysfunction (Wang et al., 2001).

Necrosis is a type of cell death that occurs without the usual programmed processes, typically triggered by severe external factors such as a lack of blood flow (ischemia), insufficient oxygen (hypoxia), or damage from physical or chemical agents, resulting in acute injury to the cells. In cases of CIRI, if the ischemic period is prolonged or if the reperfusion is excessively damaging, neuronal cells can experience necrosis. This process involves a breakdown of the cell membrane, leading to cell swelling, rupture of organelles, and the spilling of cellular contents into the surrounding space, which in turn sparks an inflammatory response in nearby tissues. Necrosis tends to happen quickly, causing immediate and significant harm to brain tissue, potentially resulting in localized brain tissue death (infarction) and softening. This can severely impair brain function and may even pose a life-threatening risk (Yuan et al., 2013).

The pathophysiology of CIRI also involves the theory of energy metabolism disorder. However, as this aspect is not the focus of this article, it will not be elaborated upon here.

### 1.2 Physiological functions of calcium ions

#### 1.2.1 Maintenance of cell membrane potential and nerve conduction

Under standard physiological circumstances, intracellular calcium ion concentrations are maintained at a relatively low level, approximately 100 nmol/L, while extracellular concentrations can escalate to between 1.2 and 1.5 mmol/L. This pronounced concentration gradient creates a potential difference across the cellular membrane, which is critical for the membrane’s optimal functioning (Chkadua et al., 2022; Beswick-Jones et al., 2023). The cell membrane is equipped with various ion channels, among which calcium ion channels are particularly vital for the conduction of nerve impulses (Altier, 2012; Gatenby and Frieden, 2017).

When nerve cells are stimulated, the first response is the opening of voltage-gated sodium ion channels in the cell membrane. This permits an influx of sodium ions into the cell, resulting in membrane depolarization and the initiation of an action potential. As this depolarization continues, voltage-gated calcium ion channels also become activated, resulting in a significant influx of calcium ions from outside the cell, driven by both concentration and electrical gradients. The calcium ions that enter the cell play a crucial role; they bind to specific proteins on neurotransmitter vesicles, which helps these vesicles move toward the presynaptic membrane (Liebeskind et al., 2012; Mokkila et al., 2017; Reva et al., 2021). Once there, the vesicles fuse with the membrane and release neurotransmitters into the synaptic cleft. These neurotransmitters then attach to their respective receptors on the postsynaptic membrane, leading to changes in the postsynaptic membrane potential and effectively transmitting the nerve impulse (Liebeskind et al., 2012; Mokkila et al., 2017; Reva et al., 2021).

An abnormal increase or decrease in the concentration of calcium ions within a cell can significantly disrupt nerve conduction. When there is an excess of calcium ions, it can trigger an excessive release of neurotransmitters, leading to heightened excitability of the nervous system. This over-excitation may manifest as symptoms like muscle spasms and seizures. On the other hand, if the concentration of calcium ions is too low, the release of neurotransmitters is suppressed, which diminishes the excitability of the nervous system. This reduction can result in problems such as muscle weakness and decreased sensory perception (Bukharaeva et al., 2022; Zhang and Stewart, 2025).

#### 1.2.2 Participation in the regulation of the cytoskeleton

Calcium ions are crucial in regulating the cytoskeleton, primarily by interacting with cytoskeletal binding proteins like tubulin and actin. This interaction influences the assembly and disassembly of microtubules and actin filaments, which in turn affects cell shape, movement, and migration (Wang and Li, 2007). Additionally, calcium ions bind to calmodulin (CaM), activating downstream effector proteins such as the microtubule-associated protein tau and calcium/calmodulin-dependent protein kinase (CaMK). This activation indirectly regulates the stability and dynamic changes of the cytoskeleton. Furthermore, calcium ions can activate various enzymes, including phospholipases, proteases, and nucleases, which can degrade cytoskeletal proteins or regulate the activity of calcium pumps, thereby influencing cytoskeletal stability (Wang et al., 2005; Zhao et al., 2006). In the context of mechanosensation, calcium ions serve as key signaling molecules that help assemble the cytoskeleton in response to external mechanical signals through their binding to CaM. During apoptosis, they play a role in remodeling the cytoskeleton by regulating microtubule arrangement and gene expression. Calcium ions also interact with the endoplasmic reticulum to manage calcium release and cellular signaling, further impacting the dynamic changes of the cytoskeleton. In processes like cell migration and invasion, calcium ions regulate the remodeling of the cytoskeleton and activate Rho family small GTPases, which control the dynamic changes of the cytoskeleton and influence cell movement across membranes. In summary, calcium ions maintain the dynamic balance of the cytoskeleton and ensure normal cell function through various mechanisms. However, under pathological conditions, dysregulation of calcium can lead to cytoskeletal destruction, resulting in cellular dysfunction and tissue damage (O'Connell et al., 2011; Bae et al., 2013).

#### 1.2.3 Regulation of enzyme activity and cell signal transduction

Calcium ions play a crucial role as activators of various enzymes in the human body, significantly influencing cellular physiological processes through the regulation of enzyme activity (Nguyen and Rosenzweig, 2002). One key calcium-binding protein found within cells is CaM, which has four binding sites for calcium ions (Novak et al., 2007). When the concentration of intracellular calcium ions (Ca^2+^) rises, these ions bind to CaM, resulting in the formation of the Ca^2+^-CaM complex. This complex is essential for activating several enzymes, including adenylate cyclase, guanylate cyclase, phosphodiesterase, and calcium/CaMK. For instance, adenylate cyclase catalyzes the conversion of ATP to cyclic adenosine monophosphate (cAMP), a vital second messenger that plays a significant role in regulating various cellular processes such as metabolism, cell proliferation, and differentiation (Zayzafoon, 2006; Ghosh and Jana, 2021). Once the Ca^2+^-CaM complex activates adenylate cyclase, it leads to an increase in intracellular cAMP levels, which subsequently activates protein kinase A (PKA) (Saljic et al., 2019). PKA then modulates the activity of various proteins through phosphorylation, thereby exerting control over essential cellular functions (del Pilar Gomez and Nasi, 2005; Prasad and Ramakant, 2025).

Calcium ions are essential players in cell signal transduction pathways. When extracellular signals like hormones, neurotransmitters, and growth factors attach to receptors on the cell membrane, they trigger pathways such as phospholipase C (PLC). This activation leads to the breakdown of phosphatidylinositol 4,5-bisphosphate (PIP2) in the cell membrane into two important molecules: diacylglycerol (DAG) and inositol trisphosphate (IP3) (Putney, 2001). Among these, IP3 is a water-soluble second messenger that interacts with IP3 receptors located on the endoplasmic reticulum. This interaction causes the release of stored calcium ions (Ca^2+^), resulting in a swift rise in the concentration of intracellular Ca^2+^(Lorenzon and Beam, 2008). The increased levels of Ca^2+^ can directly activate specific protein kinases, such as protein kinase C (PKC), which are crucial for various cellular processes, including cell growth, differentiation, and programmed cell death (apoptosis). Additionally, Ca^2+^ can bind to CaM, leading to the activation of calcium/CaMK. This kinase plays a significant role in cell signaling and the regulation of gene expression by phosphorylating different substrate proteins (Ochoa et al., 2003).

#### 1.2.4 Regulation of mitochondrial function

Calcium ions serve as crucial signaling molecules within cells and play a vital role in regulating various cellular functions through their interaction with mitochondria. Mitochondria, often referred to as the energy factories of the cell, can absorb and release calcium ions via transport proteins such as the mitochondrial calcium uniporter (MCU), the mitochondrial calcium/H^+^ antiporter (LETM1), and the mitochondrial sodium/calcium exchanger (NCLX) (Sun et al., 2023). This process is essential for transmitting calcium signals and regulating metabolism within the cell. When calcium ions enter the mitochondria, they activate key enzymes known as mitochondrial dehydrogenases, including pyruvate dehydrogenase (PDH), isocitrate dehydrogenase (IDH), and α-ketoglutarate dehydrogenase (OGDH). This activation promotes the tricarboxylic acid (TCA) cycle and oxidative phosphorylation, which are critical for meeting the cell’s ATP demands. Furthermore, calcium ions play a significant role in apoptosis by influencing the mitochondrial membrane potential and the activity of the mitochondrial permeability transition pore (PTP) (Chang and Zou, 2025). For instance, an overload of calcium can decrease the mitochondrial membrane potential, activate the PTP, and lead to the release of cytochrome C, thereby triggering apoptosis. In terms of energy metabolism, calcium ions quickly respond to the energy needs of the cell by regulating mitochondrial metabolism; for example, in brown adipose tissue, calcium ions interact with uncoupling protein 1 (UCP1) to promote uncoupled respiration and thermogenesis. Under conditions of cellular stress, such as hypoxia and oxidative stress, calcium ions help maintain cell survival by regulating mitochondrial function. However, excessive calcium uptake can result in mitochondrial dysfunction and ultimately lead to apoptosis. In conclusion, calcium ions regulate mitochondrial function through various mechanisms, including metabolism, membrane potential, and apoptosis, which are crucial for maintaining cellular physiological functions and responding to stress (Zhou et al., 2021).

#### 1.2.5 Other physiological functions

Calcium ions, known as coagulation factor IV, are essential in the coagulation process, playing a crucial role in both the intrinsic and extrinsic pathways (Rezaie, 2001; Gustavsson and Han, 2009; Anitua et al., 2021). When blood vessels are damaged, the exposed tissue factor (TF) binds with coagulation factor VII in the bloodstream to create the TF-VIIa complex, which then activates coagulation factor X, converting it to Xa. In this process, calcium ions (Ca^2+^) serve as important facilitators, enhancing the interaction between various coagulation factors and improving the overall efficiency of the coagulation response. As coagulation continues, Ca^2+^ is also involved in forming prothrombin activator and in the hydrolysis of fibrinogen by thrombin, ultimately leading to blood clotting and thrombus formation, which is vital for hemostasis. In addition to their role in coagulation, calcium ions are also critical in hormone secretion from numerous endocrine cells. For instance, the secretion of insulin from pancreatic β-cells is closely linked to calcium ions. When blood glucose levels rise, glucose enters the pancreatic β-cells, resulting in an increased ATP/ADP ratio through various metabolic processes (Hoang Do and Thorn, 2015; Fletcher et al., 2018; Ma et al., 2019a; Gil-Rivera et al., 2021). This change causes potassium ion channels on the cell membrane to close, leading to depolarization of the membrane. The depolarization then activates voltage-gated calcium ion channels, allowing Ca^2+^ to flow into the cells, which raises the intracellular Ca^2+^ concentration (Enklaar et al., 2010; Bawa and Abbott, 2011; Fridlyand and Phillipson, 2011; Groschner et al., 2014; Fedlaoui et al., 2025). This increase triggers the fusion of insulin secretion granules with the cell membrane, releasing insulin into the extracellular space and helping to regulate blood glucose levels. Furthermore, calcium ions also play a role in the secretion of thyroid hormones from thyroid cells and adrenaline from adrenal medullary cells, contributing to the maintenance of internal environmental stability and normal physiological functions by regulating these hormones’ secretion (Braun et al., 2009; Uchida et al., 2011; Omar-Hmeadi and Idevall-Hagren, 2021; Zhuang et al., 2023).

### 1.3 The role of calcium ions in brain ischemic injury

#### 1.3.1 Dynamic Changes of Calcium Ions in Brain Cells During Cerebral Ischemia-reperfusion

**FIGURE 1:**
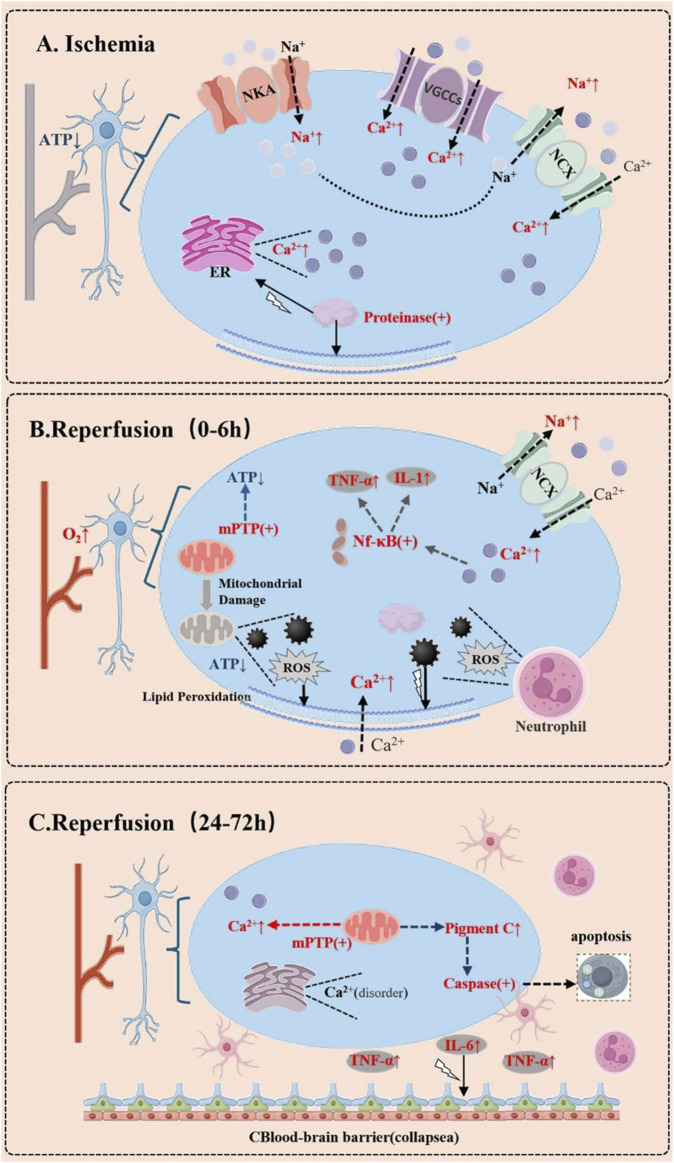
Dynamic Changes of Calcium Ions in Brain Cells During Cerebral Ischemia-Reperfusion. **(A)** During the Ischemic Phase. Due to energy depletion, the dysfunction of ion pumps on the cell membrane leads to intracellular sodium ion (Na^+^) accumulation. This triggers the reverse operation of the sodium-calcium exchanger (NCX), resulting in a massive influx of calcium ions (Ca^2+^). Additionally, the opening of voltage-gated calcium channels (VGCCs) and the release of intracellular calcium stores (e.g., the endoplasmic reticulum, ER) further cause a sharp rise in cytosolic calcium concentration. This activates a cascade of enzymes, such as proteases, phospholipases, and endonucleases, leading to structural damage and apoptosis. **(B)** During the Early Reperfusion Phase (0–6 h). Calcium dysregulation may continue to worsen. Although reperfusion restores oxygen and nutrient supply, it also induces mitochondrial dysfunction, further releasing calcium ions. The increased production of reactive oxygen species (ROS) disrupts cell membrane integrity, exacerbating calcium influx. Concurrently, the inflammatory response is initiated, characterized by neutrophil infiltration and the release of inflammatory cytokines, which may further disrupt calcium homeostasis. **(C)** In the Delayed Phase (24–72 h). Persistent calcium overload may lead to prolonged opening of the mitochondrial permeability transition pore (mPTP), triggering apoptosis or necrosis. Simultaneously, the inflammatory response intensifies, with activated glial cells releasing more inflammatory mediators, which may further destabilize calcium ion balance.

During cerebral ischemia-reperfusion, calcium ions undergo significant fluctuations in various types of brain cells (Figure 1) (Wang et al., 2013; Sharma et al., 2024a; Sharma et al., 2024b). Research indicates that under ischemic conditions, there is a marked increase in calcium ion concentration in both neurons and glial cells, with this calcium overload being a critical factor contributing to cell death (Yan et al., 2012; Lee et al., 2013). Specifically, ischemia activates T-type calcium channels, leading to a swift accumulation of intracellular calcium ions, which in turn causes excitotoxicity and apoptosis (Park et al., 2013; Liu and Sang, 2024). When blood flow is restored during the reperfusion phase, the influx of calcium ions intensifies, particularly in neurons, resulting in even greater cell damage. Furthermore, glial cells also experience notable changes in calcium ion levels during ischemia-reperfusion, and these fluctuations not only impact glial cell function but may also affect the survival of nearby neurons through intercellular signaling. Consequently, managing calcium ion homeostasis, particularly in the acute phase of ischemia-reperfusion, could offer new therapeutic avenues for protecting brain cells (Fang et al., 2022; Yaghoobi et al., 2024).

#### 1.3.2 Calcium ion imbalance and cell injury

Under normal physiological conditions, nerve cells maintain a delicate balance of intracellular calcium ions through specialized ion channels and pumps located on their membranes, ensuring that calcium ion concentrations remain very low (Fang et al., 2022; Song et al., 2024). However, during cerebral ischemia, a disruption in energy metabolism leads to a rapid decline in intracellular ATP levels, which impairs the function of ATP-dependent ion pumps, such as the sodium-potassium pump (Na^+^-K^+^-ATPase) and the calcium pump (Ca^2+^-ATPase) (Zhang et al., 2014; Milbourn et al., 2017; Zaretsky and Zaretskaia, 2020). This impairment prevents the effective removal of Na^+^ from the cell, resulting in an influx of extracellular Ca^2+^ driven by concentration and electrical gradients. Additionally, ischemia induces depolarization of the cell membrane, which activates voltage-gated calcium channels, further increasing the influx of Ca^2+^(Hirabayashi et al., 2016; Baev et al., 2017). Moreover, ischemic conditions can trigger the release of Ca^2+^ from intracellular stores, such as the endoplasmic reticulum, worsening the situation by contributing to intracellular calcium overload. This overload can directly harm cells, as excessively high levels of calcium ions increase the permeability of the cell membrane (Hu et al., 2015; Ľupták and Hroudová, 2019). This increased permeability allows intracellular substances to leak out while permitting harmful extracellular substances to enter, thereby disrupting the cell’s normal structure and function. Furthermore, excessive intracellular calcium can lead to mitochondria absorbing too much Ca^2+^, which disrupts their normal operations. This interference results in a decrease in mitochondrial membrane potential, energy metabolism disturbances, and an increase in the production of reactive oxygen species (ROS). Since mitochondria serve as the cell’s energy factories, their dysfunction significantly exacerbates cell injury and can ultimately lead to cell death (Yonutas et al., 2016; Vekaria et al., 2017; Chang and Zou, 2025; Iba et al., 2025).

#### 1.3.3 Activation of intracellular signaling pathways

When the concentration of calcium ions inside cells rises, it triggers a cascade of intracellular signaling pathways, with the activation of phospholipases, proteases, and nucleases playing a crucial role in causing cell damage. Calcium ions can activate specific phospholipases, including phospholipase A2 (PLA2) and phospholipase C (PLC). Once PLA2 is activated, it breaks down phospholipids in the cell membrane, resulting in the production of arachidonic acid (AA) and lysophospholipids. AA, an unsaturated fatty acid, can be further metabolized to create inflammatory mediators like prostaglandins and leukotrienes, which trigger inflammatory responses that lead to vasodilation, increased permeability, and the infiltration of leukocytes, ultimately worsening brain tissue damage (McHowat and Creer, 2004). Additionally, lysophospholipids are highly cytotoxic; they can compromise the structure and function of the cell membrane, reducing its stability, causing the loss of intracellular substances, and further disrupting the normal physiological functions of the cell. When PLC is activated, it hydrolyzes phosphatidylinositol-4,5-bisphosphate (PIP2) in the cell membrane into diacylglycerol (DAG) and inositol trisphosphate (IP3). IP3 binds to receptors on the endoplasmic reticulum, promoting the release of stored calcium ions (Ca^2+^), which exacerbates the overload of intracellular calcium. Meanwhile, DAG activates PKC, a serine/threonine protein kinase that phosphorylates various substrate proteins and is involved in processes such as cell proliferation, differentiation, and apoptosis (Touyz and Schiffrin, 2001; O'Neill et al., 2003). In the context of brain ischemia-reperfusion injury, excessive PKC activation can disrupt intracellular signaling pathways, leading to a series of pathophysiological changes that promote inflammatory responses, increase oxidative stress, and induce cell apoptosis, thereby intensifying neuronal damage.

Calcium ions play a crucial role in activating proteases, notably calpain, which is a calcium-dependent cysteine protease. Under normal conditions, calpain’s activity is tightly regulated; however, when there is an overload of intracellular calcium, calpain becomes excessively activated. This overactivation allows calpain to hydrolyze various proteins within the cell, including cytoskeletal proteins, membrane proteins, and enzyme proteins. The hydrolysis of cytoskeletal proteins can lead to significant changes in cell shape and the destruction of the cytoskeletal structure, which in turn affects the cell’s normal functions and stability (Romo-Mancillas et al., 2019). Additionally, the hydrolysis of membrane proteins can compromise the integrity and functionality of the cell membrane, increasing its permeability. This change can cause the loss of intracellular substances and allow harmful external substances to enter the cell. Furthermore, when enzyme proteins are hydrolyzed, it can result in the loss of activity of various enzymes, disrupting the cell’s metabolic and signaling processes, ultimately leading to cell damage and death. Similarly, calcium ions can activate nucleases, such as endonucleases. In the event of intracellular calcium overload, endonucleases become activated and can cleave DNA, resulting in DNA strand breaks. Since DNA carries the genetic information of cells, any damage to it can lead to abnormal gene expression, which disrupts normal physiological functions and the cell’s ability to proliferate and differentiate. Severe DNA damage can even trigger the apoptotic program, culminating in cell death (Matsunaga et al., 2009).

#### 1.3.4 The relationship between free radicals and calcium

The interaction between free radicals and calcium ions in cells plays a crucial role in various pathological processes, particularly in conditions like CIRI. Free radicals, which include reactive oxygen species (ROS) and reactive nitrogen species (RNS), are highly reactive molecules capable of damaging cell membranes, proteins, and DNA, ultimately impairing cellular structure and function (Cheung, 2003; Valko et al., 2007). Calcium ions serve as vital intracellular second messengers that regulate numerous cellular functions, such as muscle contraction, nerve conduction, and cell division (Bracci et al., 2002). Under normal circumstances, calcium ion concentrations within cells are carefully controlled to ensure cellular balance. However, during pathological events like ischemia-reperfusion injury, the overproduction of free radicals can lead to lipid peroxidation of cell membranes (Scatena, 2012; Chen et al., 2022a). This process compromises the integrity of the cell membrane and disrupts the normal regulatory mechanisms governing calcium ions. As a result, there can be an excessive influx of calcium ions, leading to an abnormal increase in intracellular calcium levels. This surge triggers a cascade of detrimental cellular responses, including mitochondrial dysfunction, altered enzyme activity, and damage to the cytoskeleton (Prasad Panda and Kesharwani, 2023).

Calcium overload can lead to the production of free radicals, creating a harmful cycle. For instance, when calcium ions are elevated, they can activate specific enzymes like phospholipases and proteases (Adalbert et al., 2002; García-Rivas and Torre-Amione, 2009). The activation of these enzymes results in additional cellular damage and an increase in free radical production. Additionally, calcium ions can directly influence mitochondria, disrupting their electron transport chain and further enhancing free radical generation. Consequently, the interplay between free radicals and calcium ions is mutually reinforcing, with both contributing to the processes of cellular injury and death in a synergistic manner (Sun et al., 2015).

#### 1.3.5 Inflammatory response and immune regulation

Calcium ions are crucial in activating inflammatory cells and releasing inflammatory mediators, which significantly influence the inflammatory response during CIRI (Mabon et al., 2000; Hernandez Pichardo et al., 2023). In this context, inflammatory cells like neutrophils, monocytes, and macrophages become activated and gather at the injury site. Their activation is closely linked to calcium ions. When these cells encounter conditions such as ischemia, hypoxia, and oxidative stress, the ion channels in their membranes undergo changes, resulting in an increased influx of calcium ions (Kaufmann et al., 2022; Alizadehasl et al., 2024). This rise in intracellular calcium concentration triggers a cascade of signaling pathways that enhance the activation and function of these inflammatory cells. For instance, in neutrophils, the influx of calcium ions activates pathways like PKC and mitogen-activated protein kinase (MAPK), leading to the expression and release of various inflammatory mediators, including TNF-α, IL-1β, and IL-6. TNF-α is a significant pro-inflammatory cytokine that can activate other inflammatory cells, intensify the inflammatory response, and induce apoptosis, which directly damages nerve cells. Similarly, IL-1β and IL-6 exhibit strong pro-inflammatory effects; they promote the chemotaxis, adhesion, and activation of inflammatory cells, worsening the inflammatory response and contributing to further brain tissue damage (Buck and Pamenter, 2006; Chen et al., 2010; Beermann et al., 2015).

Calcium ions play a crucial role in regulating the movement and adhesion of inflammatory cells. On the surface of these cells, various adhesion molecules, including integrins and selectins, are present, and their expression and activity are influenced by calcium ions (Sayah et al., 2003; Chang et al., 2010). When the concentration of intracellular calcium ions rises, it enhances the expression and activation of these adhesion molecules, facilitating the adhesion of inflammatory cells to the vascular endothelial cells (Shen et al., 2001; Hiraiwa, 2003; Kaczmarek et al., 2005). This process allows them to traverse the vessel wall and infiltrate brain tissue, which can amplify the inflammatory response. Furthermore, calcium ions also impact the phagocytic capabilities of inflammatory cells, boosting their ability to engulf pathogens and dead tissue, which may inadvertently harm normal tissue. In the context of immune regulation, calcium ions are essential for the activation and functional regulation of immune cells, particularly T lymphocytes and B lymphocytes, which are vital components of the immune system. Their activation and proliferation depend on calcium ions. In cases of CIRI, the abnormal activation of immune cells can lead to dysregulated immune responses, worsening damage to brain tissue. Calcium ions modulate the function of these immune cells by influencing their signaling pathways, thereby playing a significant role in the inflammatory response and immune regulation during CIRI (Brown, 2001; Yilmaz and Granger, 2010; Barkauskas et al., 2013; Sala et al., 2023).

#### 1.3.6 Induction of cell apoptosis and necrosis

Calcium ions play a significant role in inducing cell apoptosis and necrosis during CIRI through various mechanisms, with the mitochondrial pathway and endoplasmic reticulum stress being two crucial pathways. Mitochondria are central to the process of apoptosis, and an overload of calcium is a key factor that leads to mitochondrial dysfunction and the initiation of apoptosis (Kumar et al., 2012; Li et al., 2022a; Shen and Zhan, 2022). During cerebral ischemia, there is an accumulation of intracellular calcium, resulting in excessive calcium ions entering the mitochondria (Kristián, 2004; Tuo et al., 2022; Rahi and Kaundal, 2024). This overload of calcium ions causes a decrease in mitochondrial membrane potential and the opening of the mitochondrial permeability transition pore (mPTP), which is a non-specific channel situated between the inner and outer mitochondrial membranes (He et al., 2008; Fedotcheva and Fedotcheva, 2021; Endlicher et al., 2023). Under normal conditions, the mPTP remains closed, but it opens in response to stimuli such as calcium overload and oxidative stress. Once the mPTP opens, the mitochondrial membrane potential is compromised, respiratory chain function is impaired, ATP synthesis is reduced, and cellular energy metabolism is disrupted. Additionally, mitochondria release various pro-apoptotic factors, including cytochrome C (Cyt C), apoptosis-inducing factor (AIF), and endonuclease G (Endo G). When Cyt C is released into the cytoplasm, it binds to apoptosome-activating factor-1 (Apaf-1) and caspase-9 (Caspase-9) to form the apoptosome, which activates Caspase-9. This activation subsequently triggers downstream effector caspases, such as Caspase-3, leading to a cascade reaction of apoptosis and ultimately resulting in cell death. Meanwhile, AIF and Endo G can directly enter the nucleus, causing DNA fragmentation and chromatin condensation, which further promotes the occurrence of apoptosis.

The endoplasmic reticulum (ER) serves as a crucial site for intracellular calcium storage and protein synthesis, playing a vital role in maintaining calcium balance within cells and ensuring the proper folding and modification of proteins (Lu et al., 2022). In cases of CIRI, an overload of intracellular calcium can trigger stress within the endoplasmic reticulum (Hetz et al., 2020; Chen et al., 2022b; Wu et al., 2022). This stress occurs when the ER’s ability to process protein folding and modification is compromised, resulting in the accumulation of unfolded or misfolded proteins. Such a buildup initiates a series of stress responses aimed at restoring normal function (Shen et al., 2004; Wang and Kaufman, 2016; Chong et al., 2017). When the ER experiences stress, it activates the unfolded protein response (UPR) to help regain its functionality. The UPR manages cellular physiological processes through three main signaling pathways: the protein kinase R-like endoplasmic reticulum kinase (PERK) pathway, the inositol-requiring enzyme 1 (IRE1) pathway, and the activating transcription factor 6 (ATF6) pathway (Gupta et al., 2015; Wang et al., 2021a; Wang et al., 2021b; Ong et al., 2024a). However, in situations where calcium overload causes ER stress, excessive activation of these pathways can lead to cell death or apoptosis (Guan et al., 2014; Kim et al., 2015; Prentice et al., 2015). For instance, the PERK pathway, once activated, phosphorylates eukaryotic initiation factor 2α (eIF2α), which inhibits protein synthesis and helps reduce the accumulation of unfolded proteins (Iwawaki and Akai, 2006; Cui et al., 2011; Teske et al., 2011). If ER stress persists, it results in the upregulation of transcription factors like ATF4 and CHOP, with CHOP promoting the expression of pro-apoptotic genes such as Bim and PUMA. The proteins produced by these pro-apoptotic genes can either activate the mitochondrial apoptotic pathway or directly affect the cell membrane and organelles, ultimately leading to apoptosis.

Upon activation of the IRE1 pathway, it cleaves the mRNA of X-box binding protein 1 (XBP1), resulting in the production of the active XBP1s protein, which plays a crucial role in the adaptive response to endoplasmic reticulum (ER) stress (Cross et al., 2012; Jheng et al., 2012; Xu et al., 2021). When ER stress is severe, IRE1 can also trigger signaling pathways like c-Jun N-terminal kinase (JNK). This pathway can phosphorylate pro-apoptotic proteins such as Bax and Bak, which belong to the Bcl-2 family. The activation of these proteins leads to their insertion into the mitochondrial membrane, increasing its permeability. This change allows pro-apoptotic factors to be released, ultimately inducing apoptosis. Beyond the mitochondrial pathway and ER stress, calcium ions can also promote apoptosis and necrosis through various mechanisms, including the activation of death receptor pathways and the regulation of intracellular redox states. In the context of CIRI, these processes are interconnected and collaborate, resulting in the apoptosis and necrosis of nerve cells, which further aggravates brain tissue damage (Ozbal et al., 2008; Guo and Lu, 2024).

#### 1.3.7 Coagulation system and calcium

Ischemia-reperfusion injury can cause significant damage to endothelial cells, which leads to the exposure of subendothelial collagen and the activation of the coagulation cascade. This process results in the upregulation of tissue factor (TF), which activates the extrinsic coagulation pathway, leading to increased thrombin generation, fibrin deposition, and the formation of microthrombi. When the coagulation system is excessively activated, it can result in thrombosis within the microvasculature, causing disturbances in the microcirculation that further worsen tissue ischemia and hypoxia, creating a vicious cycle (Yu et al., 2018; Yang et al., 2019). Calcium ions are crucial cofactors in the coagulation cascade, necessary for the activation of several coagulation factors, including factor IX, X, and VII; without calcium ions, the coagulation process cannot function properly. Additionally, calcium ions are vital for platelet activation and aggregation. During ischemia-reperfusion, there is an influx of calcium ions that promotes platelet activation and the release of pro-coagulant substances like ADP and thromboxane A2, which further intensifies coagulation and the formation of microthrombi (Perino, 2020). An overload of calcium can lead to endothelial cell dysfunction, increased vascular permeability, and the release of tissue factor, thereby activating the coagulation system. In summary, during ischemia-reperfusion injury, calcium ions and the coagulation system interact in complex ways that exacerbate tissue damage. Calcium ions enhance the activity of the coagulation system by promoting cell injury, inflammatory responses, and platelet activation, while the activation of the coagulation system further aggravates calcium overload and tissue damage through the formation of microthrombi and inflammatory responses (Ding et al., 2000; Aliotta et al., 2021; Yu et al., 2023).

#### 1.3.8 The interplay of lipid rafts and calcium signaling in brain ischemia-reperfusion injury

In cerebral ischemia,the interaction between lipid rafts and calcium ions (Ca^2+^) significantly exacerbates ischemic injury by regulating neuroinflammatory signaling, cell death pathways, and blood-brain barrier disruption. The triggers of lipid raft and calcium signaling dysregulation in cerebral ischemia include energy metabolism crisis and oxidative stress. Ischemia-induced ATP depletion leads to dysfunction of Na^+^/K^+^-ATPase and Ca^2+^-ATPase, resulting in intracellular calcium overload (Capozzi et al., 2023; Riitano et al., 2023). The stability of lipid rafts is also impaired, as disrupted cholesterol metabolism (e.g., sphingomyelin hydrolysis) causes structural disintegration of lipid rafts, affecting the localization of membrane receptors and calcium channels (de Almeida et al., 2003; Capozzi et al., 2023). Simultaneously, oxidative stress promotes free radical attack on unsaturated fatty acids and cholesterol within lipid rafts, altering membrane fluidity and inducing hyperactivation of calcium influx channels such as NMDA receptors and TRPM2 (Stulnig et al., 2001; Díaz et al., 2024; Ong et al., 2024b). Furthermore, lipid rafts and calcium synergistically drive ischemic neuroinflammation: DAMPs (e.g., HMGB1, ATP) released post-ischemia cluster TLR4 in lipid rafts, activating the TLR4/MyD88/NF-κB pathway to trigger inflammatory signaling. Calcium signaling enhances NF-κB nuclear translocation via STIM1/Orai1 and TRPM7, amplifying the release of TNF-α and IL-1β (Kong et al., 2016; Capozzi et al., 2023). Experimental studies demonstrate that cholesterol depletion (e.g., using statins) or calcium antagonists (e.g., nimodipine) can inhibit microglial activation. Additionally, calcium influx through P2X7 receptors or mitochondrial calcium overload activates the NLRP3 inflammasome, driving IL-1β maturation, while lipid rafts provide a platform for NLRP3 assembly. Post-ischemic glutamate surges activate NMDA receptors clustered in lipid rafts, causing lethal calcium influx and neuronal death, and interventions targeting raft cholesterol (e.g., statins) reduce NMDA receptor hyperactivation (Besshoh et al., 2005; Miguel, 2025). Blood-brain barrier disruption involves calcium-dependent MMP-9 activation in lipid rafts, which degrades tight junction proteins (e.g., occludin), while VEGF induces calcium oscillations via VEGFR2 in lipid rafts, increasing vascular permeability (Lochhead et al., 2010; Muthusamy et al., 2014). Key molecular mechanisms involve positive feedback loops: ischemia-triggered DAMPs activate P2X7 receptors in lipid rafts, increasing calcium influx. Calcium-dependent enzymes further destabilize membranes, exacerbate oxidative stress, and amplify lipid raft damage. Inflammatory cytokines also feedback to upregulate TLR and calcium channel expression in rafts. Mitochondrial calcium overload generates ROS, oxidizing lipid rafts and opening more calcium channels, forming a vicious cycle. Therapeutic strategies targeting lipid rafts (e.g., statins, sphingomyelinase inhibitors) stabilize rafts and protect the blood-brain barrier, while SOCE inhibitors and TRP channel antagonists show potential in alleviating microglial inflammation. Combination therapies (e.g., antioxidants with calcium regulators) exhibit neuroprotective efficacy in clinical trials. Future research should focus on dynamic imaging of lipid raft-calcium interactions and cell-specific targeting (e.g., microglial lipid raft signaling) to develop precise therapeutic interventions (Silva et al., 2009; Thorstenberg et al., 2020; Capozzi et al., 2023; Zhong et al., 2024).

#### 1.3.9 Heparanase and calcium

Heparanase, an enzyme that cleaves heparan sulfate proteoglycans, has been implicated in various pathological processes, including endothelial damage (Rops et al., 2004; Pape et al., 2021). This damage is often characterized by increased permeability and inflammation, which can significantly affect vascular integrity and function (Wang et al., 2020; Wang et al., 2021a; Wakasugi et al., 2024). Recent studies have suggested that calcium ions (Ca^2+^) play a critical role in modulating the effects of heparanase on endothelial cells (Matzner et al., 1990; Godder et al., 1991; Zhang et al., 2013).

Calcium ions are essential for numerous cellular processes, including signal transduction, gene expression, and cell adhesion (Denhardt et al., 1995; Tran et al., 2018). In the context of endothelial damage, Ca^2+^ can influence the activity of heparanase through various mechanisms. For instance, elevated intracellular calcium levels have been shown to activate certain signaling pathways that exacerbate endothelial dysfunction (Perrier et al., 2009; Seeley et al., 2013; Lu et al., 2021). These pathways may lead to the upregulation of heparanase expression or activity, thereby contributing to increased endothelial permeability and inflammatio (Wang et al., 2016; Lukasz et al., 2017; Xu et al., 2017).

Moreover, calcium ions can also affect the structural integrity of endothelial cell junctions. The disruption of these junctions, often mediated by heparanase, facilitates the extravasation of leukocytes and plasma proteins, further aggravating tissue injury. Understanding the interplay between heparanase and calcium ions is crucial, as it may provide insights into potential therapeutic targets for conditions characterized by endothelial dysfunction, such as atherosclerosis and diabetic vascular complications. In conclusion, the association between heparanase and endothelial damage is complex and is potentially modulated by calcium ions. Further research is warranted to elucidate the precise mechanisms involved and to explore the therapeutic implications of targeting this pathway in vascular diseases.

#### 1.3.10 Calcium and wingless/integrated (WNT) signaling pathway

The WNT signaling pathway plays a crucial role in cell biology, particularly in processes such as cell proliferation, differentiation, and migration (Zhao et al., 2022a). As members of the low-density lipoprotein receptor-related protein family, LRP6 and LRP8 are involved in the regulation of the WNT signaling pathway (Roslan et al., 2019). LRP6 is considered one of the core receptors of the WNT signaling pathway; its activation can promote the accumulation of β-catenin, thereby regulating the expression of downstream genes (Zhao et al., 2022b). In contrast, LRP8 plays an auxiliary role in WNT signal transduction, potentially influencing cellular behavior by modulating intracellular calcium ion concentrations.

Research indicates that the activation of WNT signaling can lead to an increase in intracellular calcium ion concentration, potentially through the activation of the phosphatidylinositol metabolic pathway (Slusarski et al., 1997; Gong et al., 2017). Calcium ions, as signaling molecules, can affect various cellular processes, including cell proliferation, migration, and differentiation. Therefore, the activation of LRP6 not only participates in WNT signal transduction but may also influence cellular functions by modulating calcium ion concentrations (Berendsen et al., 2011; Ramachandran et al., 2018; Zhao et al., 2022a).

Furthermore, calcium ions can also provide feedback regulation on the activity of the WNT pathway by affecting intracellular signaling molecules, thereby further enhancing or inhibiting the transmission of WNT signals (Kühl, 2004; Slusarski and Pelegri, 2007; Lu and Carson, 2009). This complex interplay suggests that LRP8 and LRP6 not only function as receptors in the WNT signaling pathway but are also closely related to the dynamic changes of calcium ions.

Therefore, future research could further explore the specific mechanisms by which LRP8 and LRP6 regulate calcium ion concentrations in the WNT signaling pathway and the biological significance of this regulation in different physiological and pathological states. This will help us better understand the multifunctional roles of the WNT signaling pathway and its implications in disease.

### 1.4 Treatment strategies related to calcium ions

#### 1.4.1 Application of calcium channel blockers

Calcium channel blockers are a group of medications that specifically target calcium ion channels on cell membranes, effectively preventing the entry of extracellular calcium ions and consequently lowering the concentration of intracellular calcium ions (Makioka et al., 2001; Frishman, 2007; Makani et al., 2011; Nimmrich and Eckert, 2013; Takenaka et al., 2013; Qiu et al., 2023). These drugs hold considerable promise in treating CIRI. Among them, nimodipine is a widely used dihydropyridine calcium channel blocker in clinical settings, recognized for its high lipophilicity, which enables it to cross the blood-brain barrier with ease (Monzani et al., 2015). This property allows nimodipine to selectively dilate cerebral blood vessels and enhance cerebral blood flow, thereby offering protective benefits to brain tissue affected by ischemia (Hu et al., 2003; Xu et al., 2008). The drug works mainly by blocking L-type calcium channels located on the membranes of smooth muscle cells in the cerebral vasculature (Chaplin and Amberg, 2012). This blockade inhibits the influx of calcium ions, resulting in the relaxation of vascular smooth muscle and subsequent vasodilation, which improves blood circulation in the brain and helps mitigate the effects of CIRI. In cases of cerebral vasospasm following subarachnoid hemorrhage, nimodipine has proven effective in alleviating vascular spasms, reducing neurological damage, and enhancing patient outcomes (Kiser, 2014). Numerous clinical studies have demonstrated that administering nimodipine early can significantly lower both disability and mortality rates in patients who have experienced subarachnoid hemorrhage (de Oliveira Manoel and Macdonald, 2018; Dayyani et al., 2022; Hao et al., 2022).

Flunarizine, also known as Flunarizine Hydrochloride, is a selective calcium ion antagonist that effectively dilates capillaries and helps prevent damage caused by elevated intracellular calcium ions during ischemia and hypoxia. By blocking calcium channels on cell membranes, flunarizine reduces calcium ion influx, which inhibits the contraction of vascular smooth muscle, leading to blood vessel dilation and increased cerebral blood flow. Additionally, it can inhibit platelet aggregation, lower blood viscosity, and enhance microcirculation, demonstrating significant efficacy for symptoms arising from insufficient blood supply to the vertebrobasilar artery and migraines. In cases of CIRI, flunarizine has been shown to alleviate neuronal damage and improve neurological function. For instance, a study involving patients with acute cerebral infarction revealed that those treated with flunarizine experienced a notable decrease in neurological deficit scores and an improvement in their daily living abilities. However, while calcium channel blockers like flunarizine have shown some effectiveness in treating CIRI, they also come with limitations. Some patients may experience side effects such as dizziness, headaches, facial flushing, palpitations, and hypotension, which can impact patient compliance and overall treatment outcomes. Furthermore, the therapeutic effects of calcium channel blockers may be limited in cases where brain tissue has already sustained severe cell damage and death. It is also important to note that different types of calcium channel blockers can vary in their efficacy and safety profiles, necessitating that clinicians carefully select appropriate medications and dosages based on the individual patient’s condition (Liu et al., 2000; Berger et al., 2002; Gulati et al., 2015).

The perioperative management value of calcium channel blockers (CCBs), such as diltiazem, in carotid endarterectomy (CEA) and cardiopulmonary bypass surgery has garnered increasing attention, particularly in high-risk patients with comorbid stroke. Studies indicate that perioperative use of CCBs can effectively reduce the incidence of postoperative complications, including cardiac events and neurocognitive deficits (Park et al., 2009; Haley et al., 2021).

In cardiopulmonary bypass surgery, monitoring calcium levels is a critical step to prevent neurocognitive deficits. Calcium stability is essential for maintaining normal neurological function, especially when managing conditions that may lead to cerebral hypoperfusion. Intraoperative calcium monitoring enables timely identification of calcium fluctuations, allowing for adjustments in medication strategies to ensure stable cerebral blood flow (Conrad et al., 2013). Additionally, perioperative hypertension control is recognized as a key strategy for mitigating stroke risk. Rational use of CCBs may help achieve this goal to some extent (Anderson et al., 2007).

For postoperative monitoring of CEA patients, potential side effects of CCBs—such as hypotension and heart rate variability—must be closely observed, as these may impact recovery. Furthermore, research highlights that perioperative drug selection should account for the patient’s overall health status and tolerance to CCBs to optimize therapeutic outcomes (Aydin and Aydin, 2019). In summary, combining perioperative CCB administration with calcium monitoring provides a safer and more effective treatment strategy for stroke patients, thereby reducing postoperative complications and improving overall prognosis (Table 1).

**TABLE 1:** Comparison of different calcium channel blockers.

Drug	Target	Mechanism of action	Advantages	Limitations
Nimodipine	L-type calcium channels	Inhibits calcium influx, dilates cerebral blood vessels	Reduces incidence of vasospasm	Hypotension, dizziness
Flunarizine	Glial cell calcium channels	Inhibits calcium overload, antioxidant effects	Improves neurological function scores	Long-term use causes extrapyramidal reactions
Esmolol	Mitochondrial calcium pump	Promotes calcium efflux, protects mitochondria	Strong antioxidant activity	Risk of blood pressure fluctuations
Diltiazem	L-type calcium channels	Inhibits calcium influx, dilates peripheral blood vessels	Effectively controls blood pressure, reduces cardiac load	Bradycardia, hypotension, constipation

#### 1.4.2 Drug development for regulating intracellular calcium homeostasis

With the in-depth study of how calcium ions contribute to brain ischemia-reperfusion injury, researchers are increasingly focusing on developing drugs that can effectively regulate intracellular calcium homeostasis (Mizuno et al., 2013; Honarnejad et al., 2014; Ge et al., 2022). This area of research is gaining momentum, as new drug candidates are being explored, offering fresh hope for treating brain ischemia-reperfusion injury (Lv et al., 2020; Li et al., 2022b; Luo et al., 2024). One promising strategy involves creating drugs that directly influence the release and uptake of calcium ions from the cell’s internal stores, particularly the endoplasmic reticulum, which serves as a crucial reservoir for calcium (Verkhratsky and Petersen, 2002; Stepanova et al., 2005; Kuum et al., 2015). By targeting calcium ion channels and transport proteins located on the endoplasmic reticulum, it is possible to control the flow of calcium ions, thereby maintaining a balanced intracellular environment. Recent studies have identified certain small molecular compounds that specifically interact with inositol trisphosphate (IP3) receptors on the endoplasmic reticulum, modulating their calcium release and presenting potential therapeutic options for brain ischemia-reperfusion injury. Another strategy focuses on regulating the function of ion exchangers and pumps on the cell membrane, such as the sodium-calcium exchanger (NCX) and the calcium pump (Ca^2+^-ATPase), which are vital for maintaining calcium balance within cells. By enhancing the activity of these proteins, particularly the calcium pump, it is possible to promote the removal of excess intracellular calcium, thus alleviating calcium overload (Garcia et al., 2001; Strehler, 2015; Xie et al., 2020). Some drugs have shown promise in boosting the activity of the calcium pump, leading to increased calcium uptake and a subsequent reduction in cytoplasmic calcium concentration, which may help mitigate the cellular damage associated with calcium overload.

Certain drugs known for their antioxidant and anti-inflammatory properties have been discovered to play a role in regulating intracellular calcium levels indirectly (Du et al., 2016; Malik et al., 2020; Mathew and Panonnummal, 2021). In cases of brain ischemia-reperfusion injury, oxidative stress and inflammation can disrupt the balance of calcium within cells. However, the use of antioxidants and anti-inflammatory medications can help mitigate these stressors, thereby offering protection to intracellular calcium homeostasis. A notable example is melatonin, an endogenous hormone recognized for its antioxidant and anti-inflammatory effects. Research indicates that melatonin can help alleviate acute necrotizing pancreatitis by influencing calcium balance within cells. This may occur through mechanisms such as directly inhibiting the entry of calcium ions or facilitating the release of calcium from within cells, which in turn reduces the harmful effects associated with elevated intracellular calcium levels (Fatherazi et al., 2003; Sun et al., 2012; Abdoul-Azize et al., 2017). At present, many of these calcium-regulating drugs remain in the research phase. While some promising outcomes have been observed in animal studies, there is still a significant gap before these treatments can be applied in clinical settings (Zgavc et al., 2011; van Hout et al., 2016; Stanton et al., 2024). The drug development process necessitates further exploration into the mechanisms of action, safety, and effectiveness of these medications, along with addressing challenges related to drug targeting and pharmacokinetics to enhance their therapeutic potential and safety profiles (Hemperly et al., 2018; Vermeire et al., 2023; Viscusi et al., 2024).

#### 1.4.3 Combined treatment strategies

The use of calcium channel blockers or other calcium-regulating drugs alone may present certain limitations when addressing CIRI, (Sobrado et al., 2003). Recently, there has been a growing interest in combined treatment strategies, as pairing calcium channel blockers with antioxidants, anti-inflammatory drugs, and other agents can harness the synergistic effects of these medications to improve therapeutic outcomes. The combination of calcium channel blockers and antioxidants offers notable benefits (Miljanich and Ramachandran, 1995; Vogel, 2006; Farah and Shurtz-Swirski, 2008). In cases of CIRI, oxidative stress and calcium overload are two critical mechanisms of injury that interact, creating a vicious cycle that worsens damage to brain tissue. Calcium channel blockers help mitigate calcium overload by inhibiting the influx of calcium ions, while antioxidants work to neutralize excess free radicals in the body, thereby reducing oxidative stress damage. By using both treatments together, it is possible to simultaneously address these two injury mechanisms, leading to more effective protection of brain tissue (Du et al., 2009; Fang et al., 2020; Yang et al., 2022). Research has demonstrated that the combination of nimodipine and vitamin E significantly reduces neurological deficits in rats suffering from CIRI, lowers MDA levels in brain tissue, and enhances SOD activity, suggesting that combined treatment can boost antioxidant capacity and diminish oxidative stress damage (Rael et al., 2019).

The combination of calcium channel blockers and anti-inflammatory drugs demonstrates promising therapeutic effects, particularly in the context of CIRI, where the inflammatory response significantly contributes to nerve cell damage and death. Calcium channel blockers enhance cerebral blood circulation and help alleviate ischemic-hypoxic damage, while anti-inflammatory drugs work to inhibit the activation of inflammatory cells and the release of inflammatory mediators, thereby reducing the overall inflammatory response (Ma et al., 2019b; Fan and Lei, 2022). When used together, these medications can address CIRI from multiple perspectives. For instance, the pairing of nimodipine and ibuprofen has been shown to decrease the levels of inflammatory mediators like TNF-α and IL-1β in the brain tissue of rats suffering from this condition, leading to a reduction in inflammation and an improvement in neurological function (Detsi et al., 2007; Wu et al., 2014). Beyond their combination with antioxidants and anti-inflammatory agents, calcium channel blockers can also be effectively paired with other types of medications, such as neurotrophic factors and antiplatelet drugs. Neurotrophic factors support the survival, growth, and differentiation of nerve cells, and when combined with calcium channel blockers, they can facilitate the repair and regeneration of damaged nerve cells. Similarly, antiplatelet drugs help prevent thrombosis by inhibiting platelet aggregation, and their use alongside calcium channel blockers can enhance cerebral microcirculation, thereby reducing the risk of CIRI. Ongoing research is focused on refining treatment protocols that involve the combination of calcium channel blockers with other drugs for the management of CIRI. In clinical settings, it is essential for healthcare providers to thoughtfully select combined treatment strategies tailored to the individual patient’s condition to optimize therapeutic outcomes and improve prognosis (Jiang et al., 2010; AbouAitah et al., 2021).

#### 1.4.4 Other potential treatment methods

With the continuous advancement of medical technology, emerging treatment methods like gene therapy and cell therapy have demonstrated significant potential in regulating calcium ion-related mechanisms, offering new hope for treating CIRI. Gene therapy involves altering gene expression to treat diseases. In the context of CIRI, it can target calcium ion-related signaling pathways and proteins. By employing gene transfection technology to introduce genes that encode calcium channel regulatory proteins into nerve cells, it may be possible to modulate the function of calcium channels on the cell membrane. This modulation could reduce calcium ion influx, thereby alleviating the damage caused by calcium overload in the cells. Additionally, gene editing technology can repair or knock out gene mutations that disrupt calcium homeostasis, addressing the fundamental issue of intracellular calcium imbalance. Although research on gene therapy for cerebral ischemia-reperfusion in CIRI jury is still in the experimental phase and faces challenges such as ensuring the safety and targeting of gene vectors and regulating gene expression, it presents a promising avenue for future treatments (Jin et al., 2001).

Cell therapy leverages the unique biological properties of cells to repair damaged tissues and organs, showing significant promise in the regulation of calcium ion-related mechanisms (Bigham et al., 2024). Stem cells, known for their ability to self-renew and differentiate into various cell types such as nerve cells and vascular endothelial cells, play a crucial role in this process (Lin and Gu, 2011; Han et al., 2019; Liu et al., 2022). When stem cells are transplanted to areas affected by CIRI, they can transform into nerve cells, effectively replacing those that have been damaged and helping to restore neural function (Li et al., 2025). Additionally, these stem cells secrete a range of neurotrophic factors and cytokines that help modulate the local microenvironment, promoting the survival and repair of nerve cells (He et al., 2023; Zou et al., 2024). The neurotrophic factors released by stem cells are particularly important as they can influence calcium ion-related signaling pathways, enhance intracellular calcium balance, and mitigate damage to nerve cells. Currently, clinical trials exploring stem cell therapy for CIRI are underway, and while some preliminary results have been promising, there is still a need for further refinement of treatment protocols to improve both efficacy and safety.

Emerging therapeutic technologies, including nanotechnology and photodynamic therapy, are being investigated for their potential to regulate calcium ion-related mechanisms and treat CIRI. Nanotechnology enables the creation of nano-drug carriers that can deliver medications directly to damaged brain tissue, which enhances drug effectiveness while minimizing side effects (Du et al., 2022). On the other hand, photodynamic therapy employs specific wavelengths of light to activate photosensitizers, leading to the production of reactive substances like singlet oxygen (Ding et al., 2015; Sztandera et al., 2022; Saita and Kawasaki, 2023). This process selectively targets and destroys diseased cells while also influencing various physiological processes within those cells, particularly calcium ion-related signaling pathways. Although these innovative treatment methods are still in the early research phase, they open up new avenues for understanding and addressing CIRI (He et al., 2022; 2024; Lyu et al., 2024). As technology continues to advance and research deepens, these approaches hold promise for improving treatment outcomes for patients suffering from this condition (Clemensen et al., 2007; Gorder et al., 2024; Schneider et al., 2024).

### 1.5 Research Outlook and challenges

#### 1.5.1 In-depth study of mechanisms

While there is a growing understanding of the role of calcium ions in CIRI, many areas remain unexplored and warrant further investigation (Liu and Sang,2024). Future research could delve into the detailed analysis of specific signaling pathways, particularly by clarifying the molecular events and regulatory mechanisms associated with calcium ion-activated phospholipases, proteases, and nucleases (Sharma et al., 2024a; Yaghoobi et al., 2024). Additionally, examining the unique functions and interactions of different subtypes of calcium ion channels in the context of CIRI could pave the way for the development of more targeted therapeutic drugs. It is recognized that various calcium ion channels, including L-type, N-type, and T-type, are involved in this injury, yet their individual mechanisms of action and contributions at different pathological stages are not fully understood (Gao et al., 2016; Sabek et al., 2025). Another critical area for future research is the identification of new molecular targets related to calcium ions. The advancement of molecular biology technologies, such as proteomics and gene editing, offers powerful tools for discovering these new targets. By utilizing these technologies, researchers can analyze changes in protein expression and modification during CIRI, screen for potential molecular targets closely linked to calcium ion homeostasis, and investigate their roles in the injury mechanism, ultimately laying the groundwork for the development of innovative therapeutic drugs (Piatek et al., 2018; Ji et al., 2021; Danaeifar and Najafi, 2024).

#### 1.5.2 Development of new therapeutic drugs

Developing more effective and safer calcium ion-related therapeutic drugs is a significant goal for future research (Yang et al., 2013; Andres et al., 2015). One of the key strategies in this endeavor is structure-based drug design, which involves analyzing the three-dimensional structures of calcium ion channels and related proteins. This analysis provides valuable insights into how drugs interact with their targets, allowing researchers to design drug molecules that exhibit greater affinity and specificity (Gandini et al., 2015; Iranpour et al., 2021; Guo et al., 2024). By utilizing advanced techniques such as X-ray crystallography and cryo-electron microscopy to obtain high-precision structures of calcium ion channels, researchers can engage in computer-aided drug design. This process helps optimize the structure of drug molecules, enhancing their selectivity and inhibitory activity toward targets while also minimizing side effects. Additionally, high-throughput screening technology plays a crucial role in the development of new drugs (Mondal et al., 2010; Ozsvari et al., 2017; Kang et al., 2024). By creating large-scale compound libraries and employing automated experimental techniques alongside high-throughput detection methods, researchers can quickly screen for compounds that show activity against calcium ion-related targets. These promising compounds can then undergo further structural optimization and activity validation, paving the way for the discovery of new drugs with potential clinical applications. Moreover, the application of artificial intelligence and machine learning methodologies to interpret and anticipate screening data can substantially improve both the efficiency and success rates of the screening endeavors (Dar et al., 2018; Tiwari et al., 2023; Kumar et al., 2025).

#### 1.5.3 Clinical translation and application

Translating basic research findings into clinical treatment methods presents a significant challenge today, primarily due to the substantial gap between laboratory research and clinical practice. This process involves multiple stages of validation and optimization, transitioning from basic research to clinical trials and ultimately to clinical application (Lippi et al., 2016; Jing and Zhu, 2023). In clinical trials, it is crucial to control various factors strictly to ensure the reliability and effectiveness of the research outcomes. The considerable individual differences and complexities of conditions in patients suffering from CIRI further complicate the development of personalized treatment plans, which is a critical issue that must be addressed during the clinical translation process (James et al., 2015; Payne et al., 2019; Bendowska et al., 2022). To enhance clinical translation, it is essential to foster collaboration between basic and clinical research by establishing multidisciplinary research teams that include experts from neuroscience, pharmacology, and clinical medicine. Conducting large-scale, multi-center clinical trials is vital to verify the safety and effectiveness of new therapeutic drugs and methods (Guo et al., 2023). Furthermore, it is important to enhance training for clinical doctors to improve their understanding and application of calcium ion-related treatment strategies, ensuring that treatment plans are implemented correctly (Dai et al., 2024a; Huang and Yuan, 2024; Luo et al., 2025).

## 2 Conclusion

Calcium ions play an indispensable role in the context of brain ischemia-reperfusion injury, as their dysregulation triggers a series of pathophysiological alterations that culminate in cellular damage, the activation of diverse signaling pathways, inflammatory responses, immune modulation, and the induction of apoptosis and necrosis. These mechanisms profoundly affect the onset and progression of brain ischemia-reperfusion injury. Presently, therapeutic strategies that concentrate on calcium ion-related mechanisms, such as the utilization of calcium channel blockers, the design of agents aimed at modulating intracellular calcium concentrations, and the investigation of combination therapies, have demonstrated promise in addressing brain ischemia-reperfusion injury to a certain degree. Furthermore, novel therapeutic methods, including gene therapy and cell therapy, are emerging with potential clinical applications. Nonetheless, it is imperative to recognize the prevailing limitations in our comprehension of the role played by calcium ions in brain ischemia-reperfusion injury. The advancement of new therapeutic agents and their integration into clinical practice encounter significant obstacles. Looking ahead, it is crucial to conduct an in-depth exploration of the mechanisms associated with calcium ions, pinpoint more effective therapeutic targets, develop safer and more efficacious treatment options, and promote the amalgamation of basic research with clinical practice. This strategy aims to facilitate the transition from laboratory discoveries to clinical implementations, ultimately offering renewed hope to patients afflicted by brain ischemia-reperfusion injury, mitigating their rates of disability and mortality, and enhancing their overall quality of life.
